# Empirical comparison of three assessment instruments of clinical reasoning capability in 230 medical students

**DOI:** 10.1186/s12909-020-02185-3

**Published:** 2020-08-12

**Authors:** Yvonne Covin, Palma Longo, Neda Wick, Katherine Gavinski, James Wagner

**Affiliations:** 1 Department of Internal Medicine, Division of General and Hospital Medicine, UT Health San Antonio, 7703 Floyd Curl Drive, MC 7982, San Antonio, TX 78229 USA; 2 Department of Healthcare Sciences, UT Southwestern Medical Center, Dallas, TX USA; 3 Department of Pathology, UT Southwestern Medical Center, Dallas, TX USA; 4 Department of Internal Medicine, UT Southwestern Medical Center, Dallas, TX USA

**Keywords:** Clinical reasoning, Diagnostic reasoning, Medical student, Validity, Assessment

## Abstract

**Background:**

Several instruments intend to measure clinical reasoning capability, yet we lack evidence contextualizing their scores. The authors compared three clinical reasoning instruments [Clinical Reasoning Task (CRT), Patient Note Scoring rubric (PNS), and Summary Statement Assessment Rubric (SSAR)] using Messick’s convergent validity framework in pre-clinical medical students. Scores were compared to a validated clinical reasoning instrument, Clinical Data Interpretation (CDI).

**Method:**

Authors administered CDI and the first clinical case to 235 students. Sixteen randomly selected students (four from each CDI quartile) wrote a note on a second clinical case. Each note was scored with CRT, PNS, and SSAR. Final scores were compared to CDI.

**Results:**

CDI scores did not significantly correlate with any other instrument. A large, significant correlation between PNS and CRT was seen (*r* = 0.71; *p* = 0.002).

**Conclusions:**

None of the tested instruments outperformed the others when using CDI as a standard measure of clinical reasoning. Differing strengths of association between clinical reasoning instruments suggest they each measure different components of the clinical reasoning construct. The large correlation between CRT and PNS scoring suggests areas of novice clinical reasoning capability, which may not be yet captured in CDI or SSAR, which are weighted toward knowledge synthesis and hypothesis testing.

## Background

Clinical reasoning is of fundamental importance in the practice of medicine [1, 2]. Despite a great interest in measuring clinical reasoning ability [3, 4], educators still face challenges in practical application [5, 6]. Currently available clinical reasoning instruments have been validated using construct validity, where investigators offer evidence of the instrument’s ability to measure the intended topic [6]. Investigators also offer evidence of the instrument’s inter-rater reliability. However, the convergent validity of these instruments has received little attention [7–9]. That is, practical application is limited by our lack of understanding in how to compare scores across instruments. Furthermore, given disparate perspectives on clinical reasoning definitions [10–12], we need robust empiric studies to clarify the context of instrument scores. The medical educator’s ability to compare instruments is paramount in developing robust competency evaluation programs in medical training curricula [13]. Messick’s criteria offers a useful framework for studying the relationships of these instruments.

According to Messick’s criteria, validity evidence is comprised of five underlying arguments: content (“topic of interest”), response process (“rater and examinee actions’ alignment with construct”), internal structure (“reliability, item analysis, and factor analysis”), consequences (“impact of the assessment”), and convergent validity (“relationship to other variables”) [8, 14]. Convergent validity is a powerful, yet underutilized validity argument [6–8, 15]. The convergent validity argument is founded on the relationship of a novel instrument’s score to scoring of associated instruments. Two instruments measuring the same information should be strongly, positively related. Conversely, there should be little to no appreciable correlation between instruments measuring unrelated phenomena. Convergent validity studies allow the emergence of unexpected data to challenge previously held assumptions about real world observations, and theories about unobservable constructs [16]. Convergent validity does not address whether the intended construct is measured (which is content validity), but rather how similar (or dissimilar) the information captured by the new instrument is to other instruments.

This study’s purpose is to determine the convergent validity of three clinical reasoning instruments: Clinical Reasoning Task (CRT) checklist [17], Patient Note Scoring Rubric (PNS) [18], and Summary Statement Assessment Rubric (SSAR) [19], by comparing each instrument’s scoring of clinical notes created at the conclusion of a virtual patient module to Clinical Data Interpretation (CDI) test [20, 21] scores. Moreover, to further evaluate real world associations, we investigated the relationships of each instrument’s scoring to student characteristics.

## Method

### Data collection

**Fig. 1:**
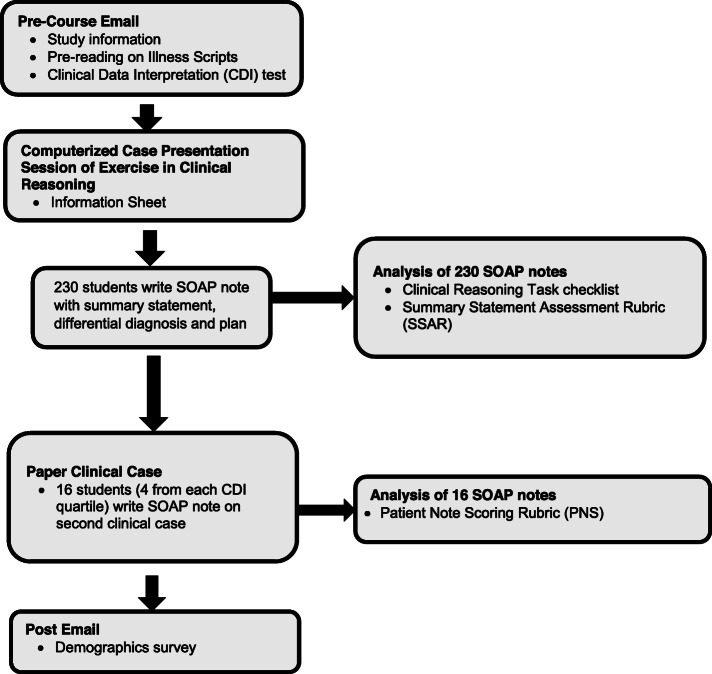
Comparison of Three Assessment Instruments of Clinical Reasoning Capability

In November 2016, at the end their 18-month pre-clinical curriculum, 235 students began a two-week *Foundations of Clinical Reasoning* course at a large academic medical center in the United States. All data collection took place on the first day of the *Foundations of Clinical Reasoning* course [Fig. 1]. Students completed the CDI test prior to the first session. Students worked in small groups on a computer-based case presentation of an *Exercise in Clinical Reasoning* [22]*.* The case paused twice for students to input a working differential diagnosis and plan. At the conclusion of the case, each student wrote an individual clinical note. We randomly-selected four students from each CDI quartile (*n* = 16) to write a clinical note on a second published clinical case [23].

Demographic variables included potential associations with novice clinical reasoning ability: college major, premedical clinical experiences (e.g. medical volunteering, apprenticeships, health professions careers), gender, and self-reported ethnicity [24, 25]. No exclusion criteria applied. Students were not incentivized for participation.

### Instruments

The Clinical Data Interpretation (CDI) test is a 72-item multiple-choice question instrument. The CDI is grounded in script concordance theory, and seeks to determine clinical reasoning capability during diagnostic uncertainty [21]. In the same template as the Script Concordance Test [26, 27], for each item, after considering a chief complaint and one item of clinical data, students designate a likelihood for a diagnostic hypothesis [20, 27]. In the only divergence from the Script Concordance Test, Williams and colleagues created a scoring key with a single correct answer per item [21]. Each question correct answer receives a full point. Twenty-six symptoms correlated to diagnoses across multiple clinical specialties appear in on the test. Two of these symptoms formed the presenting chief complaints for the *Exercise in Clinical Reasoning* (shortness of breath), and the second clinical case (memory loss). Each student had 60 min to complete the CDI. Raw scores are total points earned out of 72.

The Clinical Reasoning Task checklist (CRT) was developed as a taxonomy of 24 tasks physicians use to reason through clinical cases [17]. These tasks have been used to explore the reasoning patterns used by medical students, residents and attending physicians [28, 29]. Students earned one point each time a CRT task was used, including repeats, in accordance with previously published protocols [29, 30]. Total scores were assigned by adding the total number of CRT tasks used. The Patient Note Scoring Rubric (PNS) was created to capture student clinical reasoning capability [18]. The authors used the standardized scoring instrument, which covers three domains scored on a scale of 1–4 points: documentation of pertinent history and exam findings, differential diagnosis, and diagnostic workup. The Summary Statement Assessment Rubric (SSAR) is a 5-domain instrument to validated to evaluate the clinical reasoning documented within summary statements created by medical students [19]**.** The domains include factual accuracy, appropriate narrowing of the differential diagnosis, transformation of information, semantic qualifier use, and a global rating. Students received 0–2 points on each domain of their summary statement, except factual accuracy, which received 0 (inaccurate) or 1 (factually accurate).

### Analysis

Three teams of reviewers scored the clinical notes with CRT, PNS, and SSAR instruments. The primary investigator (Y.C.) reviewed each clinical note in detail with each team and selected cases to create initial examples of scoring criteria. Each team iteratively compared scoring criteria by reviewing batches of sample notes until we agreed on scoring criteria. The remaining notes, or summary statements, were coded by the team with final scores assigned by agreement. We achieved statistically significant and high-degree agreement in all qualitative coding analysis of the clinical notes. CRT reviewers (Y.C., N.W) achieved an intraclass correlation (ICC) of 0.978. The SSAR reviewers worked in two teams with K.G.’s scores being corroborated by YC (ICC = 0.831) and JW (ICC = 0.773). PNS reviewers (J.W. and K.G) achieved an ICC of 0.781 coding the 16 clinical notes for the second case.

Descriptive statistics were calculated for CDI scores, demographics, and instrument scoring. The authors correlated each instrument’s global score to CDI with Pearson correlation. Correlation analyses were performed with each instrument’s global score, and domain scores. Spearman’s rank correlations were performed to investigate non-linear correlations. Effect sizes categorized in accordance with published standards [31]. Finally, five student demographic variables were individually compared to instrument scores with one-way ANOVA [Table 1]. Due to multiple comparisons, for all reported analysis, those achieving two-tailed *p*-value ≤0.01 were considered statistically significant. Data analyzed with SPSS (IBM, version 25, 2017, Armonk, NY). The UT Southwestern Medical Center Institutional Review Board approved this study.

**Table 1:** Demographic Characteristics of 121 Medical Students in the Foundations of Clinical Reasoning Course

Characteristic	Survey Respondents (***n*** = 121)
**Gender**	
**Male**	55 (45.5%)
**Female**	63 (52.1%)
**Prefer not to answer**	3 (2.5%)
**Ethnicity**	
**American Indian or Alaska Native**	0 (0%)
**Asian or Pacific Islander**	54 (44.6%)
**African American (Non-Hispanic)**	5 (4.1%)
**Hispanic**	6 (4.9%)
**White (Non-Hispanic)**	45 (37.2%)
**Two or more/Other**	5 (4.1%)
**I prefer not to answer**	6 (4.9%)
Pre-Medical Experiences*	
**Health Professions education or employment (Nursing, Physician Assistant, Physical Therapy, Pharmacy, ect)**	15
**Volunteering or Shadowing (inpatient)**	76
**Volunteering or Shadowing (outpatient)**	87
**Scribe**	13
**Medical Mission trip**	18
**Volunteering at a hospice center, a retirement center, or crisis center**	28
**I did not participate in any clinical activities**	6
**One or more of these**	80
Hours in Pre-Medical Experiences **	
**0–50 h (One work week)**	21 (18.3*%*)
**51–100 h (Two work weeks)**	15 (13%)
**101–150 h (Three work weeks)**	11 (9.6%)
**151–200 h (Four work weeks)**	12 (10.4%)
**1–2 months**	11 (9.6%)
**2–6 months**	16 (13.9%)
**6 months - 1 year**	10 (8.7%)
**1–2 years**	6 (5.2%)
**> 2 years**	13 (11.3%)
**College Major**	
**Biology**	64 (52.9%)
**Chemistry**	12 (9.9%)
**Computer Science/Biomedical Engineering**	5 (4.1%)
**Business/Economics**	5 (4.1%)
**Psychology**	5 (4.1%)
**Language Arts (English, Spanish, ect)**	2 (1.7%)
**Other**	28 (23.1%)

* Percentages not calculated due to multiple responses

** Six students reported not participating in Pre-Medical Experiences. The participant number is 115

## Results

The CDI test was completed by 234 of 235 students (99.6% response rate). Voluntary demographic data were collected from 121 of 235 students (51.5% response rate) [Table 1]. Women represented 52.1% (*n* = 63) of the respondents. There was no statistical difference in CDI mean (SD) score among respondents and non-respondents [44.9 (5.4) vs 43.9 (4.8); *p* = 0.35]. We compared five categories of student characteristics (i.e., gender, race/ethnicity, college major, premedical clinical experience type, and premedical clinical time) with corresponding scores on each clinical reasoning assessment instrument. We did not find any significant association between any demographic variable and the corresponding student CDI, CRT, PNS, or SSAR scores with one-way ANOVA.

**Fig. 2:**
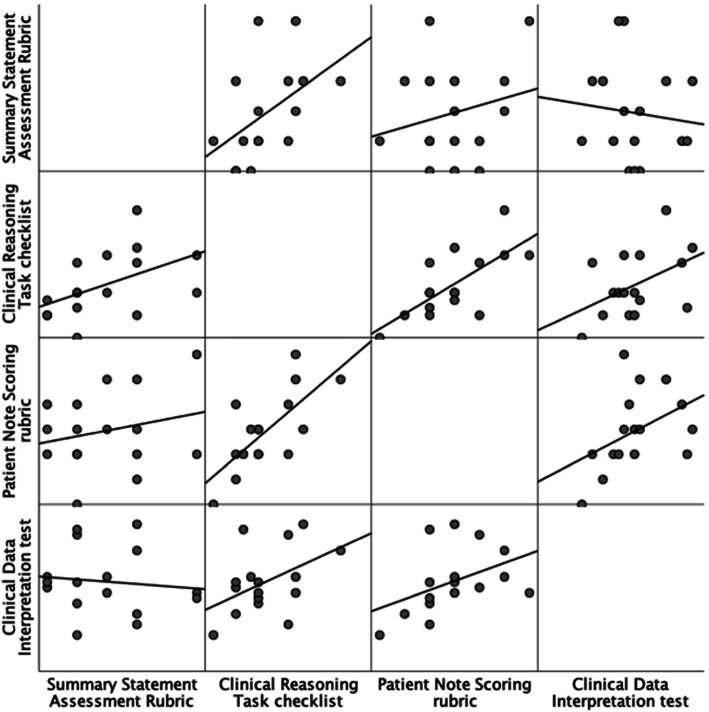
Scatterplot Matrix of Three Clinical Reasoning SOAP Note Instruments with Clinical Data Interpretation test

For the first case, 229/235 clinical notes, and 227/235 summary statements were submitted (97.5 and 96.6% response rates, respectively). All 16 students selected to submit written clinical notes from the second clinical case completed the activity. Table 2 and Fig. 2 show the Pearson product moment correlation coefficients for the correlations of the CDI score with each final clinical note score. Similar results were seen with Spearman’s Rank Correlations. The average number of CRT checklist items used per clinical note was 11.8 [range 1–30]. The mean SSAR score of the 227 summary statements was 3.68 [range 0–9]. The mean PNS score of the 16 clinical notes from the second case was 45.8 [range 24–68]. Small to medium effect sizes were seen between CDI scores and the three instruments [Table 2 and Fig. 2]. Only CRT demonstrated significance, with a small effect size with CDI (r = 0.16, *p* = 0.01, df = 228).

**Table 2:** Correlations of Three Clinical Reasoning SOAP Note Instruments with Clinical Data Interpretation score

	Clinical Reasoning Task (CRT) score (***n*** = 229)	Summary Statement Assessment Rubric (SSAR) score (***n*** = 227)	Patient Note Scoring (PNS) score (***n*** = 16)
**Clinical Data Interpretation Score (CDI test)**	r = 0.166* *p* = 0.01	r = 0.108 *p* = 0.10	r = 0.383 *p* = 0.14
**Clinical Reasoning Task score**	1	r = 0.158** *p* = 0.01	r = 0.712** *p* = 0.002
**Summary Statement Assessment Rubric score**	r = 0.158** p = 0.01	1	r = 0.145 *p* = 0.59
**Patient Note Scoring rubric score**	r = 0.712** *p* = 0.002	r = 0.145 *p* = 0.59	1

** Correlation is significant at the 0.01 level (2-tailed)

* Correlation is significant at the 0.05 level (2-tailed)

Among the three instruments, significant correlation was found between the PNS and the CRT checklist was found (r = 0.712; *p* = 0.002; df = 15) [Table 2 and Fig. 2]. To investigate the potential overlap of assessment domains between the PNS and CRT, we compared frequency of the domains within the PNS (i.e. Workup, Differential Diagnosis and Documentation) and the fourteen possible items on the CRT checklist. We observed multiple medium effect sizes and one significant correlation between these two instruments [Table 3]. Students who scored well on the PNS Documentation of history and physical exam articulated the need for consultation and follow up on the CRT (r = 0.631; *p* = 0.009; df = 15).

**Table 3:** Correlations of the components of the Patient Note Scoring and Clinical Reasoning Task instruments in 16 SOAP notes

	Documentation of History and Physical Exam	Differential Diagnosis	Workup
**Identify active issues**	r = 0.233 *p* = 0.38	r = 0.29 *p* = 0.27	r = 0.329 *p* = 0.21
**Consider alternative diagnoses and underlying cause(s)**	r = 0.219 *p* = 0.41	r = 0.091 *p* = 0.737	r = − 0.63 *p* = 0.63
**Identify precipitants or triggers to the current problem(s)**	r = 0.047 *p* = 0.86	r = − 0.291 *p* = 0.27	r = 0.052 *p* = 0.84
**Select diagnostic investigations**	r = 0.141 *p* = 0.60	r = 0.556* *p* = 0.025	r = 0.462 *p* = 0.07
**Determine the most likely diagnosis and underlying cause(s)**	r = 0.177 *p* = 0.51	r = 0.123 *p* = 0.65	r = 0.22 p = 0.41
**Identify modifiable risk factors**	r = − 0.233 *p* = 0.38	r = 0.097 *p* = 0.72	r = 0.052 p = 0.84
**Identify complications associated with the diagnosis, diagnostic investigations, or treatment**	r = − 0.184 *p* = 0.495	r = − 0.164 *p* = 0.544	r = 0.068 *p* = 0.80
**Explore physical and psychosocial consequences of the current medical conditions or treatment**	r = 0.314 *p* = 0.23	r = − 0.131 *p* = 0.62	r = 0.07 *p* = 0.79
**Establish goals of care**	r = 0.304 *p* = 0.25	r = − 0.21 *p* = 0.43	r = 0.038 *p* = 0.89
**Establish management plans**	r = 0.288 *p* = 0.27	r = 0.135 *p* = 0.61	r = 0.073 *p* = 0.78
**Determine follow-up and consultation strategies**	r = 0.631** *p* = 0.009	r = 0 *p* = 1	r = 0.561* *p* = 0.041

** Correlation is significant at the 0.01 level (2-tailed)

* Correlation is significant at the 0.05 level (2-tailed)

## Discussion

We hypothesized that the CDI test would correlate with the each of the instruments. We found a small significant correlation between CDI and CRT. The significance of this small effect size stems from a large sample size. This means that we accept our null hypothesis – none of the clinical reasoning assessment instruments demonstrated a statistically significant correlation to CDI test.

Our findings represent the first large comparison of clinical reasoning clinical note assessment instruments with a standard, the CDI test. Our results contribute to the current body of validity evidence surrounding clinical reasoning assessment instruments in the area of convergent validity, or relationship to other variables. This empirical data supports the argument that clinical reasoning is currently described by multiple theoretical frameworks that may not describe the same phenomenon [10, 11, 32]. Furthermore, our study mitigated the typical limitation of study design for convergent validity – participant time burden for multiple assessments [7] – by (a) scoring one clinical note with multiple instruments, and (b) selecting a subpopulation for a second assessment through stratifying by CDI test score.

We found a large, significant correlation between PNS and CRT global scores [Table 2], as well as specific scoring domains within each instrument [Table 3]. There is statistically significant overlap in the constructs underpinning these two instruments. These instruments may measure some of the early capabilities expressed by the novice as they communicate their clinical reasoning about a clinical case.

We hypothesized that the student characteristics selected will interact with the clinical reasoning assessment scores. We did not find statistically significant associations across the four assessment instruments. This means that educators interested in clinical reasoning assessment of clinical notes should be encouraged that the four instruments tested demonstrated objectivity across measured demographics.

Limitations of our study include that this is a single-institution study of student’s clinical reasoning capability on two written cases. Our study benefits from a large, diverse participant sample with minimal attrition. To this end, we did not find any significant associations between student demographics with variables of clinical reasoning. We rated clinical notes from a single time point, which restricted our ability to assess both temporal stability, and predictive validity of the participant scoring. To circumvent student availability in the curriculum for repeated time measurements, we did select a subpopulation of students to participate in a concurrent, separate measurement of a written (paper) clinical note after submitting their electronic clinical note. Given the large number of participants, multiple instruments, and different performance opportunities (written and electronic clinical notes), our results have significant educational impact.

Convergent validity is a powerful, yet underutilized, validity argument that serves to position, and confirm an instrument into the current understanding of the intended construct. It is accepted that there is no specific amount of validity evidence that satisfies “validity” of an instrument. Nevertheless, we propose that educators consider incorporating convergent validity in to their validation procedures, especially in conjunction with real-world scores to further evidence of extrapolation [9].

The future of clinical reasoning assessment holds promise through increasingly well-designed studies. In our study, differing strengths of association between clinical reasoning instruments suggests varying degrees of overlap in the clinical reasoning frameworks underpinning the assessment domains. Our future work will include clinical note assessment at all training levels to determine the clinical reasoning instruments most appropriate for each level of clinical reasoning capability development, and their effect on student learning as a part of a larger assessment program of this complex competency [13]. We implore researchers to include convergent validity testing when developing their instrument to situate it within the context of available assessment instruments. Such investigations will accelerate our understanding of the multidimensional construct of clinical reasoning.

## Data Availability

The datasets used and/or analyzed during the current study are available from the corresponding author on reasonable request.

## Abbreviations

CDI: Clinical Data Interpretation test
CRT: Clinical Reasoning Task checklist
ICC: Intraclass Correlation Coefficient
PNS: Patient Note Scoring rubric
SSAR: Summary Statement Assessment Rubric
